# Quantification of plasmid DNA reference materials for Shiga toxin-producing *Escherichia coli* based on UV, HR-ICP-MS and digital PCR

**DOI:** 10.1186/s13065-016-0201-0

**Published:** 2016-09-12

**Authors:** Wen Liang, Li Xu, Zhiwei Sui, Yan Li, Lanying Li, Yanli Wen, Chunhua Li, Shuzhen Ren, Gang Liu

**Affiliations:** 1 Laboratory of Biometrology, Shanghai Institute of Measurement and Testing Technology, 1500 Zhang Heng Road, Shanghai, 201203 People’s Republic of China; 2 Division of Medical and Biological Measurement, National Institute of Metrology, No.18, Bei San Huan Dong Lu, Chaoyang District, Beijing, 100013 People’s Republic of China

**Keywords:** Plasmid DNA, Reference material, Digital PCR, *Escherichia coli* O157:H7

## Abstract

**Background:**

The accuracy and metrology traceability of DNA quantification is becoming a critical theme in many fields, including diagnosis, forensic analysis, microorganism detection etc. Thus the research of DNA reference materials (RMs) and consistency of DNA quantification methods has attracted considerable research interest.

**Results:**

In this work, we developed 3 plasmid candidate RMs, containing 3 target genes of *Escherichia coli* O157:H7 (*E. coli* O157:H7) and other Shiga toxin-producing Escherichia coli (STEC): stx1, stx2, and fliC (h7) respectively. Comprehensive investigation of the plasmid RMs was performed for their sequence, purity, homogeneity and stability, and then the concentration was quantified by three different methods: ultraviolet spectrophotometer (UV), high resolution inductively coupled plasma mass spectrometry (HR-ICP-MS) and digital PCR. As a routinely applied method for DNA analysis, UV was utilized for the quantification (OD260) and purity analysis for the plasmids. HR-ICP-MS quantified the plasmid DNA through analysing the phosphorus in DNA molecules. Digital PCR distributed the DNA samples onto a microarray chip containing thousands of reaction chambers, and quantified the DNA copy numbers by analysing the number of positive signals without any calibration curves needed.

**Conclusions:**

Based on the high purification of the DNA reference materials and the optimization of dPCR analysis, we successfully achieved good consistency between UV, HR-ICP-MS and dPCR, with relative deviations lower than 10 %. We then performed the co-quantification of 3 DNA RMs with three different methods together, and the uncertainties of their concentration were evaluated. Finally, the certified values and expanded uncertainties for 3 DNA RMs (pFliC, pStx1 and pStx2) were (1.60 ± 0.10) × 10^10^ copies/μL, (1.53 ± 0.10) × 10^10^ copies/μL and (1.70 ± 0.11) × 10^10^ copies/μL respectively.

**Graphical abstract:**
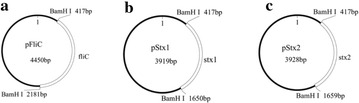
We developed 3 plasmid candidate RMs, containing 3 target genes of Escherichia coli O157:H7 (E. coli O157:H7) and other Shiga toxin-producing Escherichia coli (STEC): stx1, stx2, and fliC (h7) respectively, and the quantification of three different methods (UV, dPCR, ICP) was studied.

**Electronic supplementary material:**

The online version of this article (doi:10.1186/s13065-016-0201-0) contains supplementary material, which is available to authorized users.

## Background

Shiga-toxin-producing *Escherichia coli* (STEC) [1–3] is widely implicated to sporadic cases and serious outbreaks all over the world. Enterohemorrhagic *E. coli* O157:H7 (*E. coli* O157:H7) is one of its most threatening serotypes, which has been reported in over 30 countries causing severe infections [4]. Detection of *E. coli* O157:H7 [5] and other non O157 STEC [6] is playing a key role in diagnostics, environmental protection and food safety.

DNA analysis is taking a more and more important position in pathogenic microorganism detection, for their remarkable advantages like analysis speed, specificity, sensitivity and high-throughput. Currently, most of the DNA analysis methods are semi-quantitative, including polymerase chain reaction (PCR), sequencing, DNA chip and biosensors, most of which rely on the calibration curves or the comparing threshold value comparison. In this situation, DNA reference materials (RMs) are urgently needed, to guarantee the reliability and traceability of the quantification results. The importance of DNA RMs has been more and more highlighted for method calibration and proficiency testing. Scientists in U.S. National Institute of Standards and Technology (NIST) reported that, stable DNA quantitation RMs could obviously help to reduce the within- and among-laboratory quantitation variability [7]. However, for DNA RM [8] development, basic research of quantification methods was needed, in order to study the consistency of these methods and analyse the uncertainty sources [9].

UV spectrophotometry (UV) is commonly used for convenient DNA routine quantification by measuring the absorbance at 260 nm (OD260) [10] based on Beer–Lambert’s law for high concentration and pure DNA samples. Some other physicochemical methods have high sensitivity, accuracy and clear metrology traceability [11], however they are still often hampered by the efficiency of the phosphodiesterase enzyme digestion. HR-ICP-MS has higher sensitivity and specificity, which can accurately analyse the mass fraction of phosphorus, that stoichiometrically presents in DNA molecule [12], and consequently achieve the DNA concentration with high precision and clear traceability to the International system of units (SI) [12–15]. However, a disadvantage of UV and HR-ICP-MS is their incapability of specifically distinguishing different DNA sequences.

Real-time quantitative PCR (qPCR) is capable of sensitive and specific nucleotide acid analysis even under very low concentration. However, as a relative method, the quantification results is always traced back to UV [16], when always using a working curve. In contrast, digital PCR (dPCR) [17] as a novel promising DNA absolute quantification method, is more accurate and precise than qPCR, and most importantly, it can independently quantify DNA without calibration or internal control [18]. The sample is partitioned onto a microarray chip with thousands of separate reaction chambers, so that each chamber contains 1 or 0 target molecule. After a PCR amplification step, we can accurately determine the copy number of the original DNA samples [18, 19], by analysing the number of positive partitions (where an amplified signal is found).

When using different methods together for DNA quantification, the consistency is always a critical challenge, which seriously impedes the reliability of the results [20, 21]. Thus, it is becoming more and more important to establish method standard and DNA reference materials. In this work, we developed 3 plasmid candidate RMs for STEC analysis, containing 3 main target genes respectively: Shiga toxin 1 (stx1), Shiga toxin 2 (stx2) and flic (h7) [22]. In this work, we investigated three methods for DNA quantification: UV, HR-ICP-MS and dPCR, each of which represents a distinctive analysis principle: spectrophotometry, element assay and DNA amplification respectively. Key conditions were optimized to finally achieve improved consistency and reliable RM quantification results.

## Results

### Plasmid DNA construction

**Fig. 1:**
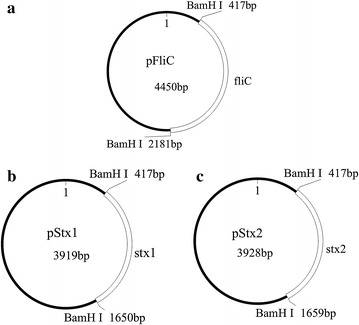
The construction maps of three plasmids: **a** pFliC, **b** pStx1, **c** pStx2

The target fragments stx1 (1227 bp), stx2 (1236 bp) and fliC (1758 bp) were ligated into pUC19 vector, and then transformed into *E. coli* JM109. The size of these recombinant plasmids were 4450 bp (pFliC), 3919 bp (pStx1), and 3928 bp (pStx2) respectively. The construction maps (Fig. 1) showed the structures of the recombinant plasmids, each of which carried a single copy of one target gene. The sequencing results confirmed that they were 100 % consistent with the initial design (data not shown).

**Fig. 2:**
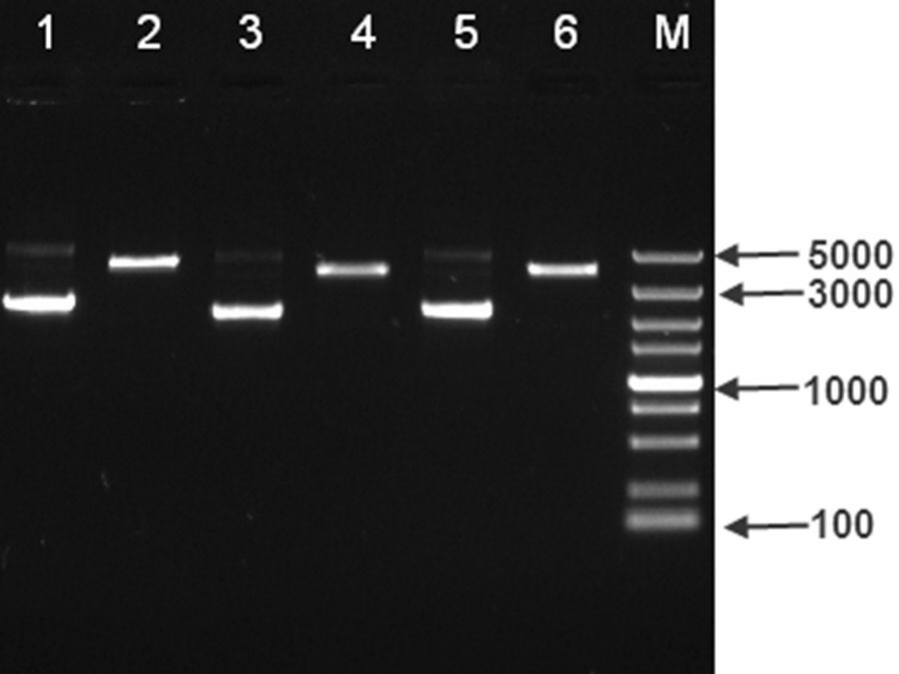
Identification of the recombinant plasmids before and after restriction digestion (RD) (*M* marker, *lane 1* pFliC before RD, *lane 2* after RD, *lane 3* pStx1 before RD, *lane 4* pStx1 after RD, *lane 5* pStx2 before RD, *lane 6* pStx2 after RD)

Then the produced plasmids were investigated by 1 % agarose gel electrophoresis. In order to eliminate the effect of the molecular secondary structures in electrophoresis, our circular plasmids were cut to linear using an enzyme digestion step. As shown in Fig. 2, the size of the digested linear plasmids was demonstrated to be the same as our design (between 3000 and 5000 bp), and no RNA and low-mass fragments were found.

For well purified double-stranded DNA (dsDNA) solution, A260/A280 should be between 1.8 and 2.0 and A260/A230 should be higher than 2.0 [23]. As our UV results showed, ratios of A260/A280 for pFliC, pStx1 and pStx2 were 1.89, 1.88 and 1.83, and ratios of A260/A230 were 2.06, 2.08 and 2.01 respectively, indicating good purity of the DNA samples (Additional file 1: Figure S1). 3 plasmids were then quantified by UV (OD260).

### Homogeneity and stability study

For each plasmid candidate RM, 300 bottles of replicates were produced, and the homogeneity of the sub-packed candidate RMs was investigated by UV. For the between-bottle homogeneity, 15 bottles of one plasmid were randomly selected from the whole batch, and 3 test portions (1 μL) from each bottle were analysed. For the within-bottle homogeneity study, 16 test portions (1 μL) from one same bottle were analysed. The sample homogeneity was assessed by a one-way analysis of variance (ANOVA) [29], and finally the F test values (Fcal) were calculated to be lower than the critical values at 95 % confidence for all 3 candidate plasmid between- and within-bottle (Additional file 1: Table S1), which clearly demonstrated their good homogeneity with the minimum sampling volume as low as 1 μL. For short-term stability study, 3 bottles of each plasmid were placed at 4 °C, for different storage time (0, 1, 3, 7 and 15 days) and quantified by UV, then the analysis data was analysed by the classic linear model and t test. The result showed that all of the three candidate RMs were stable for 15 days under 4 °C, which is adequate for the sample delivery (Additional file 1: Table S2).

The long-term stability of the candidate RMs was then continuously investigated for 12 months under −20 °C storage. One bottle of each plasmid was randomly taken out from the batch and analysed for 3 times (n = 3) by UV at 0.5, 1, 2, 4, 6, 9 and 12 months. The classic linear model and t test was used for the stability analysis. The significance factors (t) of the slopes (β1) were calculated by equation t = |β1|/s(β1), where s(β1) is the standard deviation (SD) of the slope, t represents the change of the sample concentration. Our results demonstrated 3 candidate RMs were stable in 12 months, because t was lower than t_0.95,n−2_ (under 95 % confidence level, n − 2 is the degree of freedom of data analysis (Additional file 1: Table S3).

In summary, we produced 3 plasmid candidate RMs, each of which had 300 bottles of duplicates with well demonstrated purity, homogeneity and stability.

### HR-ICP-MS quantification

HR-ICP-MS was applied to quantify the purified plasmids by analyzing the phosphorus in DNA molecules. ELEMENT2 is a double focusing magnetic sector field HR-ICP-MS, which is capable of separate possible polyatomic interferences even with a very small mass difference like ^31^P (30.97376) and 15N16O (30.99502).

**Fig. 3:**
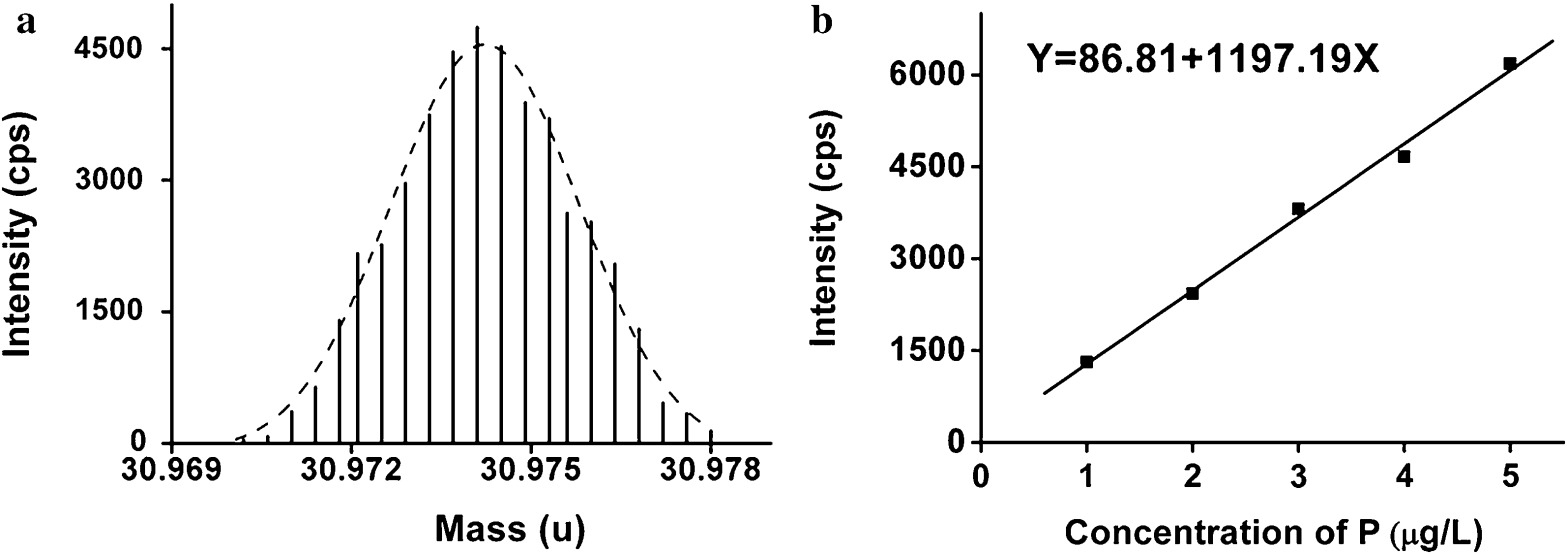
HR-ICP-MS analysis results: **a** HR-ICP-MS spectrum of phosphorus in DNA (pStx2), **b** the standard curve for phosphorus analysis using phosphorus solution certified RM (SRM3139a)

Based on the excellent resolution of HR-ICP-MS, phosphorus in DNA molecule was accurately quantified. We statistically compared the analysis results of the DNA samples before and after digestion, and the difference was demonstrated to be insignificant (Additional file 1: Figure S4) due to t-test. Thus in our work, all the HR-ICP-MS analysis was performed without any digestion treatment (Fig. 3a).

The working curve with good linear correlation was established by using a phosphorus solution certified RM (SRM3139a) from NIST (Fig. 3b). Then eight aliquots of each plasmid solution were analysed to achieve an average phosphorus concentration value. Based on P % in DNA molecules, the copy number concentrations of three plasmids were calculated.

### Digital PCR quantification

Before dPCR was performed, the primer design and sample pretreatment was investigated by qPCR. The candidate RMs were treated by restriction enzyme, and the samples before (circular DNA molecules) and after (linear DNA molecules) enzyme treatment were analysed by qPCR. The result indicated that Ct values of all the samples were delayed by 1.0–2.4 cycles without enzyme digestion, due to the inhibiting effect of the plasmids’ circular conformation [24]. Additional file 1: Figure S2 showed the qPCR result of pFliC as an example.

**Fig. 4:**
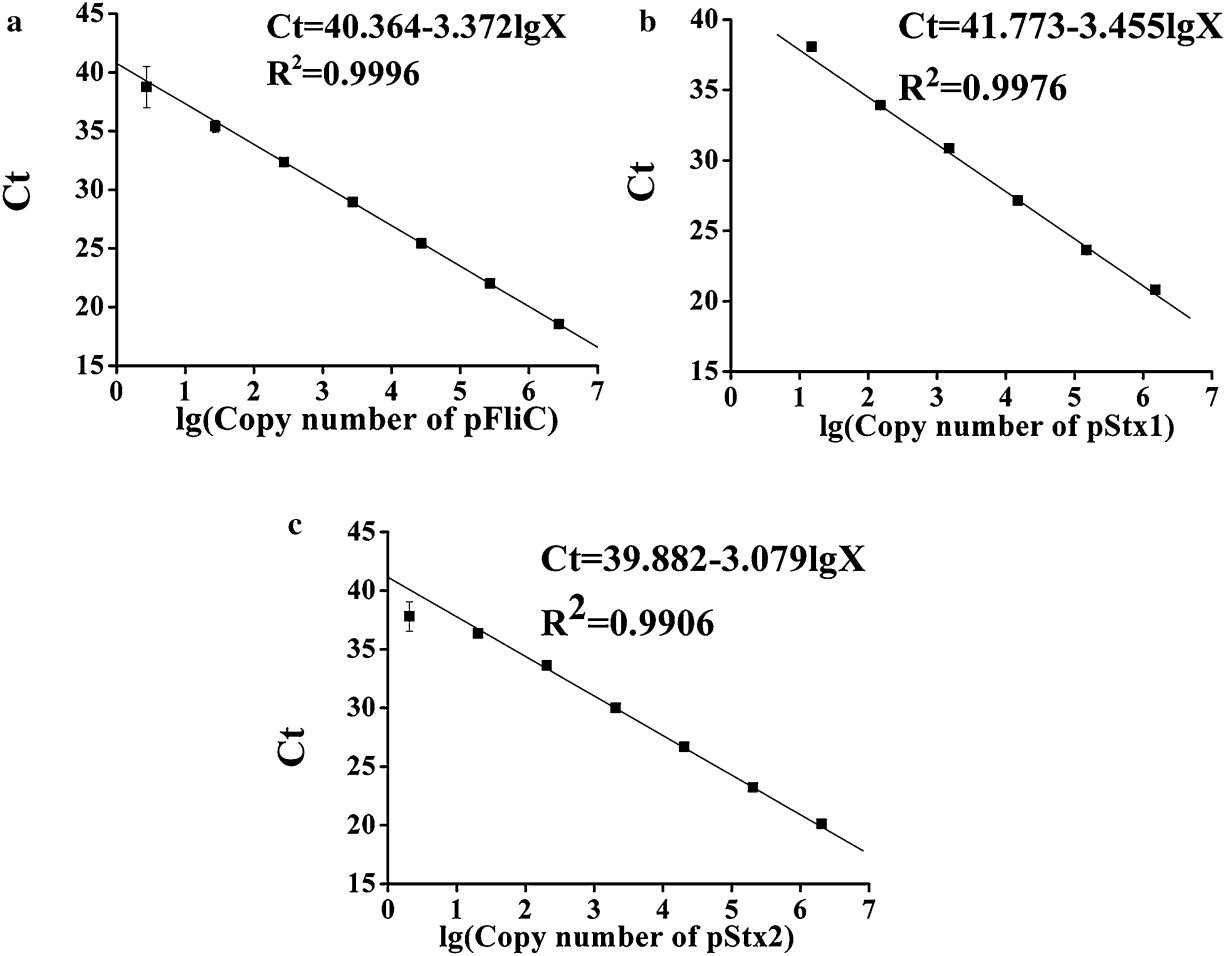
Real-time PCR calibration curves for 3 plasmid RMs: **a** pFliC, **b** pStx1 and **c** pStx2. The X was the concentration of the plasmids

We established the qPCR calibration curves for 3 genes (Fig. 4). The PCR efficiencies (E) of PCR were calculated to be between 95 and 110 % using the equation E = 10^(−1/*k*)^ − 1, where *k* is the slope of the calibration curves [25], and the R^2^ was larger than 0.99. The limit of detection (LOD) was calculated to be lower than 30 copies (3 × SD, SD was the standard deviation of the lowest Ct detected by qPCR), and no unspecific amplification was found in negative controls. These results demonstrated the validity of the plasmid production and primer designing, which was important for the following dPCR analysis.

**Fig. 5:**
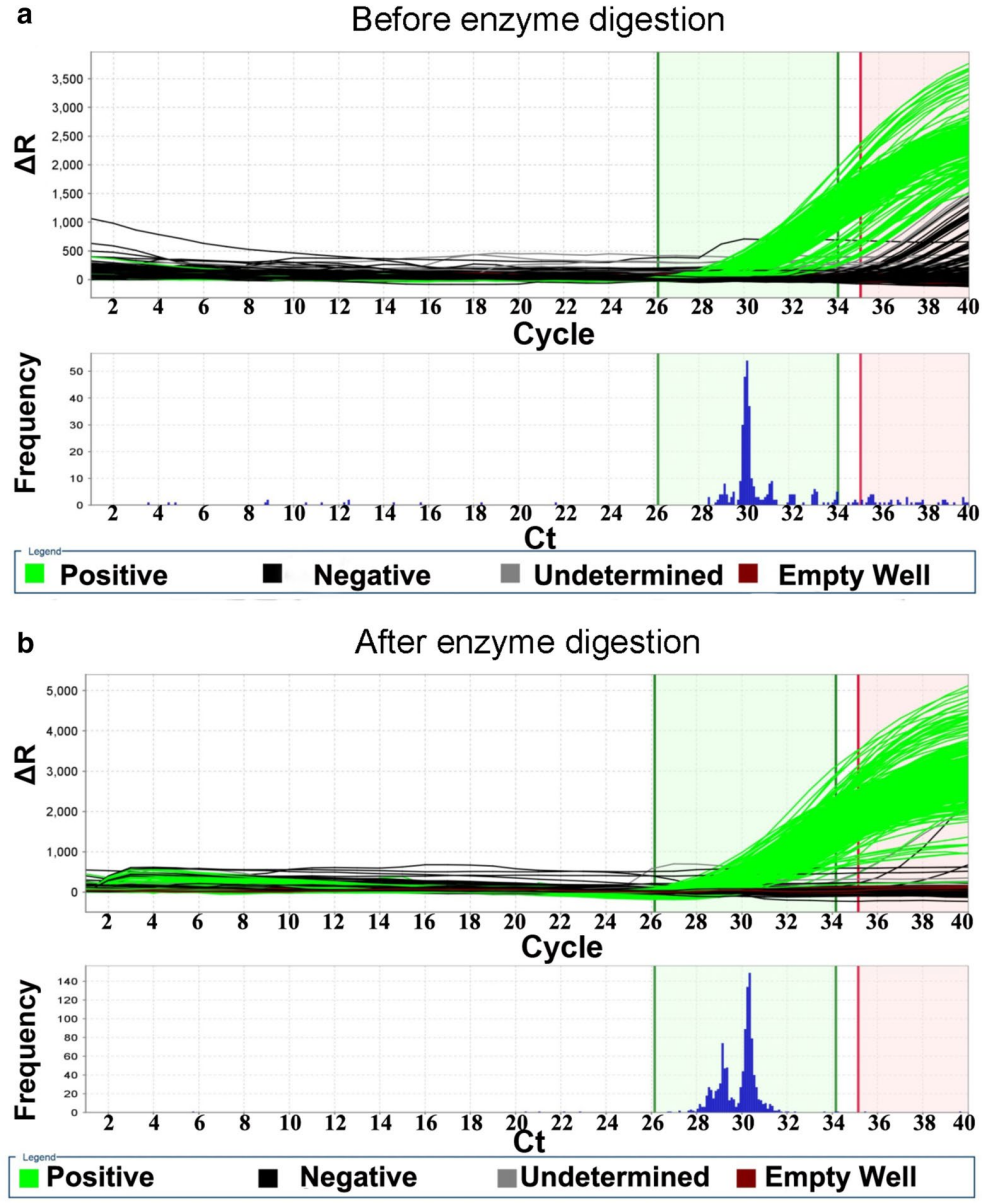
The dPCR amplification curves of pStx2 before (**a**) and after (**b**) enzyme digestion. The relative fluorescence signal (ΔR) was automatically normalized by deducting the background of each chamber. The frequency represented the number of the PCR curves with a certain Ct value

Then, dPCR was performed based on the optimized conditions. Compared to the droplet digital PCR (ddPCR) which only detects the PCR end-point fluorescence signal, the chip digital PCR in our work recorded the whole PCR amplification curves (Fig. 5) with important information of the PCR amplification, and statistically recognized positive signals based on a threshold C_rt_ value. The limit of C_rt_ values (green vertical bars in Fig. 5) were set based on 95 % confidence interval of the numbers of positive signals. The amplification curves with Ct values between the green vertical bars were taken as positive amplifications, while those beyond the limits (the red vertical bar in Fig. 5) were regarded as negative signals. Finally, thousands of adopted signals including positive and negative were analysed based on Poisson’s distribution, and reliable quantification results were achieved. When we compared the dPCR amplification curves before and after enzyme digestion (Fig. 5 was the results for pStx2), many delayed Ct values were found without enzyme digestion (black curves in Fig. 5a), causing a 53–66 % decrease of the dPCR quantification results (Additional file 1: Figure S3).

**Fig. 6:**
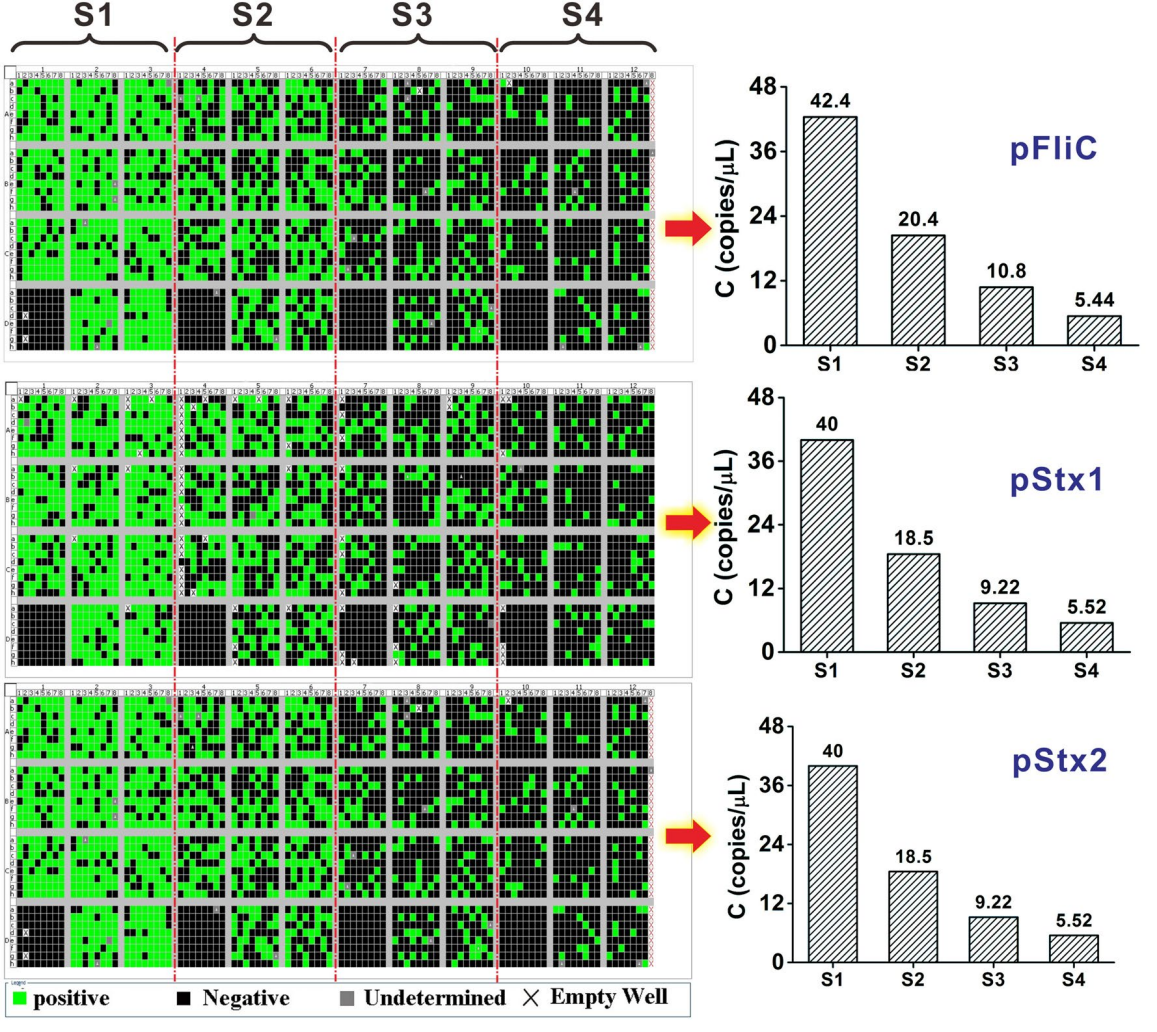
dPCR results on microarrray chips of 3 plasmids: pFliC, pStx1, pStx2 (from *top* to *bottom*). 3072 chambers on one chip were divided into 4 groups (separated by the *red dashed lines*) for 4 diluted samples (S1, S2, S3, S4). The *green points* and the *black points* represented the positive signals and the negative signals respectively, and the *gray points* were ineffective reactions due to either suspiciously abnormal baseline fluorescence or low fluorescence scores. The X marks were empty well mainly due to failed sample uploading. The *down left squares* of all the 4 groups were negative controls. *Column diagrams* on the right showed the quantification results of 4 dilutions

In dPCR quantification, totally 3072 chambers of one microarray chip were divided into 4 groups (see red dashed lines in Fig. 6), and each of the group was uploaded with a diluted concentration of the plasmid separately (from S1 to S4 in Fig. 6). Our dPCR quantification results of three plasmids were shown in Fig. 6. From left to right, the number of positive signals (green) decreased obviously with the decrease of the sample concentration. The negative controls (down left of each partition) showed no positive signals, demonstrating the absence of sample pollution or unspecific amplification.

Four quantification results from four partitions (left in Fig. 6) kept good doubling relationship, which strongly demonstrated the reliability of the dPCR quantification. Finally the average of four groups (S1–S4) was taken as the dPCR result for each plasmid.

### Method evaluation and RM certification

In order to realize reliable certification of the RM concentration and to evaluate the performance of the methods, after the quantification of 3 different methods (Additional file 1: Table S5), we assessed the uncertainty of the results, according to the internationally recognised *Guide to the expression of uncertainty in measurement (GUM)* [26], and the uncertainty was derived from two main sources:

#### (1) Uncertainty of 3 quantification methods:

Firstly, UV analysis was directly performed without sample dilution step, thus the only uncertainty source of UV result was the SD of 8 replicates (*n* = 8), the relative uncertainty of UV analysis was calculated by the equation: $u_{{c({\text{UV}})}} = {\text{SD/}}(\sqrt n \cdot \overline{{x_{\text{UV}} }} )$, where $\overline{{x_{UV} }}$ was the average concentration value achieved by UV.

Secondly, RMs were diluted to lower than 10 μg/L (by about 2000 times dilution), then analysed by HR-ICP-MS to quantify P concentration (*C*_*s*_) in the sample solution, and then DNA concentration was calculated through the $C_{{({\text{ICP}} \cdot {\text{MS}})}} = C_{s} \cdot V_{S} /(V \cdot P\,\% ),$ where *V*_*s*_ is the final sample volume, *V* is the volume of plasmid DNA RMs before dilution, and *P* % is the mass fraction of *P* element in the plasmid DNA molecules. Thus, main uncertainty sources were: first, the uncertainty of *C*_*s*_ including the SD of 8 replicates, the uncertainty of the phosphorus solution RM and the uncertainty of the standard curve fitting; Second, the uncertainty of *V*_*s*_ and *V* which came from the uncertainty of the pipettors and the volumetric flasks, and the volume change due to the temperature variation.

Thirdly, for dPCR analysis, RMs were diluted to about 18 copies/μL (by about 10^9^ times dilution), then automatically uploaded onto the microarray chips and quantified. The uncertainty sources mainly came from the dilution and the SD of 8 replicates.

**Fig. 7:**
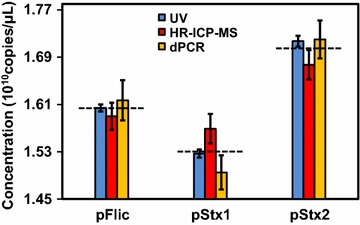
Comparison of UV (*blue column*), HR-ICP-MS (*red column*) and dPCR (*yellow column*) quantification results of three plasmid DNA RMs. The *error bars* represented the uncertainties of the methods, and the *black dashed lines* are the average concentration of the RMs

Error bars in Fig. 7 represented the uncertainties of 3 methods. The uncertainty of UV was lower than two other methods, because UV was directly performed under high concentration without any dilution. On the contrary, the RMs needed 2000 and 10^9^ times dilution before HR-ICP-MS and dPCR quantification respectively. The deep dilution operation and the lower repeatability at low concentration introduced much higher uncertainties to the final results. Owning to the optimization, we achieved very good consistence between different methods with relative deviations lower than 10 %. The black dashed lines in Fig. 7 were the average values of three RMs.

Finally, the quantification uncertainties of 3 plasmid RMs (*u*_*c*_) were calculated to be 0.052, 0.048 and 0.053 (×10^10^ copies/μL) for plasmid pFliC, pStx1 and pStx2, using the following equation:

$$ u_{c} = \bar{\bar{X}} \cdot \sqrt {\left( {\frac{{u_{c(UV)} }}{{C_{UV} }}} \right)^{2} + \left( {\frac{{u_{c(HR - ICP - MS)} }}{{C_{(HR - ICP - MS)} }}} \right)^{2} + \left( {\frac{{u_{c(dPCR)} }}{{C_{dPCR} }}} \right)^{2} } $$

#### (2) Uncertainty of the samples (instability and inhomogeneity):

Two other main uncertainty sources of the RM concentration were the uncertainty from instability (*u*_*s*_) during the storage and the uncertainty from inhomogeneity (*u*_*bb*_) between bottles. The *u*_*s*_ was calculated using the following equation: $u_{S} = {}_{S}(\beta_{1} ) \cdot X,$ where *X* is the storage time. The results were summarized in Additional file 1: Table S3. In our work, the mean square of within-bottle analysis data (*MS*_*within*_) was higher than that of between-bottle (*MS*_*between*_) (Additional file 1: Table S1), thus *u*_*bb*_ was calculated due to a simplified equation: $u_{\text{bb}} = \sqrt {MS_{\text{within}} /n} \times \sqrt[4]{{2/V_{\text{within}} }}$, where *MS*_*within*_ is the mean square of within-bottle analysis data, *n* is number of analysis replicates for one same bottle, and *V*_*within*_ is the degree of freedom of *MS*_*within*_^25^. All the data is summarized in Additional file 1: Table S1.

The uncertainties of the RM concentrations (*u*) were then summarized, including *u*_*c*_, *u*_*bb*_ and *u*_*s*_: $u = \sqrt {u_{c}^{2} + u_{\text{bb}}^{2} + u_{s}^{2} }$. The expanded uncertainty (*U*) was then calculated by multiplying *u* by the coverage factor (*k* = 2) (*U* = *k*×*u*). The detailed data of uncertainty evaluation was listed in Additional file 1: Tables S5, S6. Finally, The certified values and expanded uncertainties for plasmid pFliC, pStx1 and pStx2 were (1.60 ± 0.10) × 10^10^ copies/μL, (1.53 ± 0.10) × 10^10^ copies/μL, and (1.70 ± 0.11) × 10^10^ copies/μL respectively.

## Conclusions

The lack of DNA RMs and acknowledged DNA quantification standards, hinders the result mutual accredit and explains the significance of the consistency, uncertainty and traceability of DNA analysis methods. In this work, 3 candidate plasmid RMs for pathogenic *E. coli* detection were prepared with well investigated sequence, homogeneity and stability, and 3 different methods were studied including UV, HR-ICP-MS and dPCR, for the certification of the candidate plasmid RMs.

We investigated the accuracy and the uncertainty of all these methods. UV is simple and stable for purified and high-concentration samples, and the average relative expanded uncertainty of UV for three RMs were only 0.46 %. HR-ICP-MS has clear metrology traceability through phosphorus quantification. The diluted RMs were quantified by HR-ICP-MS, based on the high resolution of phosphorus in DNA molecules. DPCR is capable of absolute quantification of very low copy numbers of DNA based on the high-throughput microarray chips. For reliable dPCR results, enzyme digestion was researched by qPCR to achieve a distinctively improved PCR performance. However, even with high sensitivity, the complex dilution processes and environmental interferences inevitably enlarged the relative expanded uncertainty of HR-ICP-MS and dPCR to 1.52 and 1.96 % respectively.

Based on detailed optimization of 3 different methods, with very different analysis strategies and detection sensitivity, we finally combined them for the quantification of DNA reference materials. Due to the high resolution of HR-ICP-MS, we eliminated the digestion step to reduce the uncertainty of its results. Although we strongly suggest the importance of sample pretreatment for accurate dPCR to guarantee the amplifying efficiency. Another fact demonstrated by our results is that, the uncertainty from dilution steps is usually a major part, and thus the preparation of standard solutions at low concentrations should be performed very carefully, and the uncertainty should always be counted in. We choose suitable quantification methods and DNA reference materials due to the concentration level, in order to get rid of unnecessary uncertainty sources.

## Methods

### Regents and instruments

Primers and DNA sequences were synthetized by Takara (Dalian, China). *Escherichia coli* (*E. coli*) strain JM109 from Takara was used as the host for cloning and plasmid propagation. The plasmid pUC-19 vector, the restriction enzymes *Hind* III and *Sal* I, T4 DNA Polymerase and DL5000 DNA marker used in gel electrophoresis were also purchased from Takara. HR-ICP-MS was performed on the ELEMENT 2 (ThermoFisher Scientific) system. ABI QuantStudio 12K Flex qPCR System (ThermoFisher Scientific) was applied for both qPCR and dPCR (with different analysis modules). All other reagents were at least analytical grade purity. Ultrapure water (18.2 MΩ) was obtained from a Milli-Q system (Millipore).

All primers and TaqMan probes for PCR analysis were listed in Table 1.

**Table 1:** The sequences of the primers and probes for the qPCR and dPCR analysis of 3 plasmids

primer/probe	Sequence 5′–3′	Amplicon size (bp)
fliC-FP	CCGCGAGCGAAGGTAGTG	75
fliC-RP	CAGGAGTTGCTTTTGCGATAGTATAT	
fliC-probe*	FAM-CGGTGCTTCTCTGACATTCAATGGCA- TAMRA	
stx1-FP	TGCAGATAAATCGCCATTCG	123
stx1-RP	AAGCTTCAGCTGTCACAGTAACAAA	
stx1-probe	FAM-ACCTCACTGACGCAGTCTGTGGCAAGA- TAMRA	
stx2-FP	CACTGTCTGAAACTGCTCCTGTTT	78
stx2-RP	TGCTGATTCTCCCCCAGTTC	
stx2-probe	FAM-CGGTGCTTCTCTGACATTCAATGGCA- TAMRA	

* The Taqman probe was labeled with a FAM fluorophore and a TAMRA quencher

### Production of the plasmids

The sequences of three target genes were determined based on the data in GeneBank: The feature gene fliC (GenBank: AF228492.1) was a target for the detection of Serotype O157:H7 *E. coli*, while the gene stx1 (GenBank: EF079675.1) and stx2 (GenBank: GU126552.1) were specific targets for Shiga-toxin-producing *E. coli.*

We synthesized the sequences containing the target genes, each of which was flanked by two restriction *Bam*H I enzyme sites. Then the synthetic sequences were inserted into pUC19 vectors in a ligation system with a final volume of 20 μL containing 1 U T4 DNA Polymerase, Tris–acetate 33 mM, CH_3_COOK 66 mM, (CH_3_COO)_2_Mg 10 mM, DTT 0.5 mM, target gene 200 ng and vector DNA 50 ng.

The produced plasmids were then transformed into the host (JM109) mainly following a reported protocol [27], and the positive recombinant bacterial colony was confirmed by PCR reaction and DNA sequencing. Then, the recombinant bacteria was propagated in Luria–Bertani broth [10 g of tryptone, 5 g of yeast extract, and 10 g of sodium chloride in 1 L water (pH 7.4)] for 12–16 h. Bacterial cells were lysed with alkaline-SDS solution, and the produced plasmids were purified with ethanol following a reported protocol [28]. Finally, the plasmids were diluted by TE buffer (10 mM Tris, 0.1 mM EDTA, pH 7.5), and carefully divided into 300 bottles (100 μL).

### Enzyme digestion

Enzyme digestion solution was as follows: 1 μL restriction enzyme (15 U/μL), 2 μL digestion buffer, 2 μL plasmid DNA from previous step (containing about 160 ng plasmid DNA), an 15 μL H_2_O. Purified plasmid pFliC and pStx2 were digested with *Hin*dIII, and pStx1 was digested with *Sal*I. The digestion buffer for pFliC and pStx2 was as following: 100 mM Tris–HCl (pH 7.5), 100 mM MgCl_2_, 10 mM Dithiothreitol and 500 mM NaCl. The digestion buffer for plasmids pStx1 was consist of: 500 mM Tris–HCl (pH 7.5), 100 mM MgCl_2_, 10 mM Dithiothreitol and 1000 mM NaCl. The digestion was performed under 37 °C for 2 h, then the digestion result was investigated by 1 % agarose gel electrophoresis.

### UV spectrophotometry

UV quantification of the plasmids was performed on a UV–VIS spectrophotometer (Cary-100, Varian). The absorbance of the purified plasmid DNA was measured at 230, 260, 280, and 320 nm, and the mass concentration (*C*_*m*_, ng/μL) of DNA was calculated through the equation: *C*_*m*_ = (A_260_ − A_230_) × 50, where A260 and A320 are the absorbance at 260 and 320 nm, and A320 was subtracted as background absorption. For the UV quantification of the double-strand DNA (dsDNA), the molar absorption coefficients is 50 ng/µL [29]. The copy number concentration of plasmids (*C*_*c*_) was calculated using the equation: *C*_*c*_ = *C*_*m*_ × *N*_*A*_/M_DNA_, where *N*_*A*_ is the Avogadro constant, and M_DNA_ is the molecular weights of plasmids.

### HR-ICP-MS

The key conditions for HR-ICP-MS analysis were as following: the plasma power was 1350 W, the flow rate of cool gas, aux gas and sample gas was 16.86, 0.99 and 1.123 L/min respectively. A phosphorus solution RM from NIST (SRM3139a) was applied as the external standard calibration, so as to guarantee the traceability for the quantification.

The mass fraction of phosphorus (*P* %) in plasmid DNA was calculated to be 10.22 %, using the equation *P* % = 2×bp × 30.974/M_DNA_, Where bp is the number of base pairs in plasmid molecules, and M_DNA_ is the molecular weight of the plasmids. Thus, the concentration of DNA (*C*_*m*_) was achieved from the concentration of phosphorus (*P*_*mass*_) through the equation: *C*_*m*_ = *P*_*mass*_*/P* %.

### qPCR

Seven serially diluted solutions of enzyme digested plasmid DNA (with UV estimated concentration from 10^6^ to 1 copies/µL) were used to establish the PCR calibration curve. The PCR reaction mixture contained: 10 μL 2× Master Mix (TaqMan Universal PCR Master Mix with ROX reference dye pre-mixed, Applied Biosystems), 0.2 μM probe, 0.4 μM forward primer and 0.4 μM reverse primer, 1 μL enzyme digested plasmid DNA, and DNase-free H_2_O. The PCR thermal program was as follows: 95 °C for 10 min, followed by 40 cycles of 95 °C for 15 s; then 60 °C for 60 s; fluorescence was collected at the annealing and extension step (60 °C) [30]. For each analysis, the same amount of TE buffer was analyzed as the blank.

### dPCR

The thermal program, concentration of primers and probes for dPCR assay were all the same with qPCR. The dPCR chip has 3072 reaction cambers with a volume of 33 nL, and for an effective dPCR analysis, each of the chip chamber should contain 0 or 1 copy of target DNA molecule, Thus, In our experiment, four deeply diluted samples were prepared by 1 × 10^8^, 2 × 10^8^, 4 × 10^8^ and 8 × 10^8^ times (S1–S4) dilution of the original RMs. Finally, 1.25 μL of each diluted sample was added to 5 μL PCR reaction mixture and then uploaded onto microarray chips for dPCR analysis. TE buffer was analyzed as blank. After an amplification process, the dPCR curves were statistically analyzed, and the quantification results were achieved without any working standards needed.

## Additional file

10.1186/s13065-016-0201-0 Supplementary information.
