# Supplementary material for: Quantification of plasmid DNA reference materials for Shiga toxin-producing Escherichia coli based on UV, HR-ICP-MS and digital PCR
Source: Chem Cent J. 2016 Sep 12;10(1):55. doi: 10.1186/s13065-016-0201-0 (PMC5018943; doi:10.1186/s13065-016-0201-0)
Supplement: Supplementary file 1 — 10.1186/s13065-016-0201-0 Supplementary information. [file 13065_2016_201_MOESM1_ESM.doc]

**Additional Materials for:**

**Quantification of Plasmid DNA Reference Materials for *Shiga toxin-producing Escherichia coli* based on UV spectrophotometry, HR-ICP-MS and chip digital PCR**

Wen Liang ∙ Yanli Wen ∙ Li Xu ∙ Yan Li ∙ Lanying Li ∙ Di Li ∙ Chunhua Li ∙ Shuzhen Ren ∙ Gang Liu*

W Liang ∙ Y Wen ∙ L Xu ∙ Y Li ∙ LY Li ∙ CH Li ∙ SZ Ren ∙ G Liu

Laboratory of Biometrology, Shanghai Institute of Measurement and Testing Technology, 1500 Zhang Heng Road, Shanghai, PR China, 201203

E-mail: [liug@simt.com.cn](mailto:liug@simt.com.cn)

D Li

Division of Physical Biology & Bioimaging Center, Shanghai Institute of Applied Physics. Chinese Academy of Sciences, Shanghai, PR China 201800

**a**

**b**

**c**

**Fig. S1** UV spectrum of the 3 plasmids DNA RMs: pFlic (a), pStx1 (b), pStx2 (c)

**Fig. S2** qPCR analysis of diluted plasmid DNA pFliC (from left to right: 1.5E6 to 15 copies/μL). The black columns were before enzyme digestion and the white ones were after digestion

**Fig. S3** UV evaluation and dPCR results of plasmid DNA RMs before and after enzyme digestion


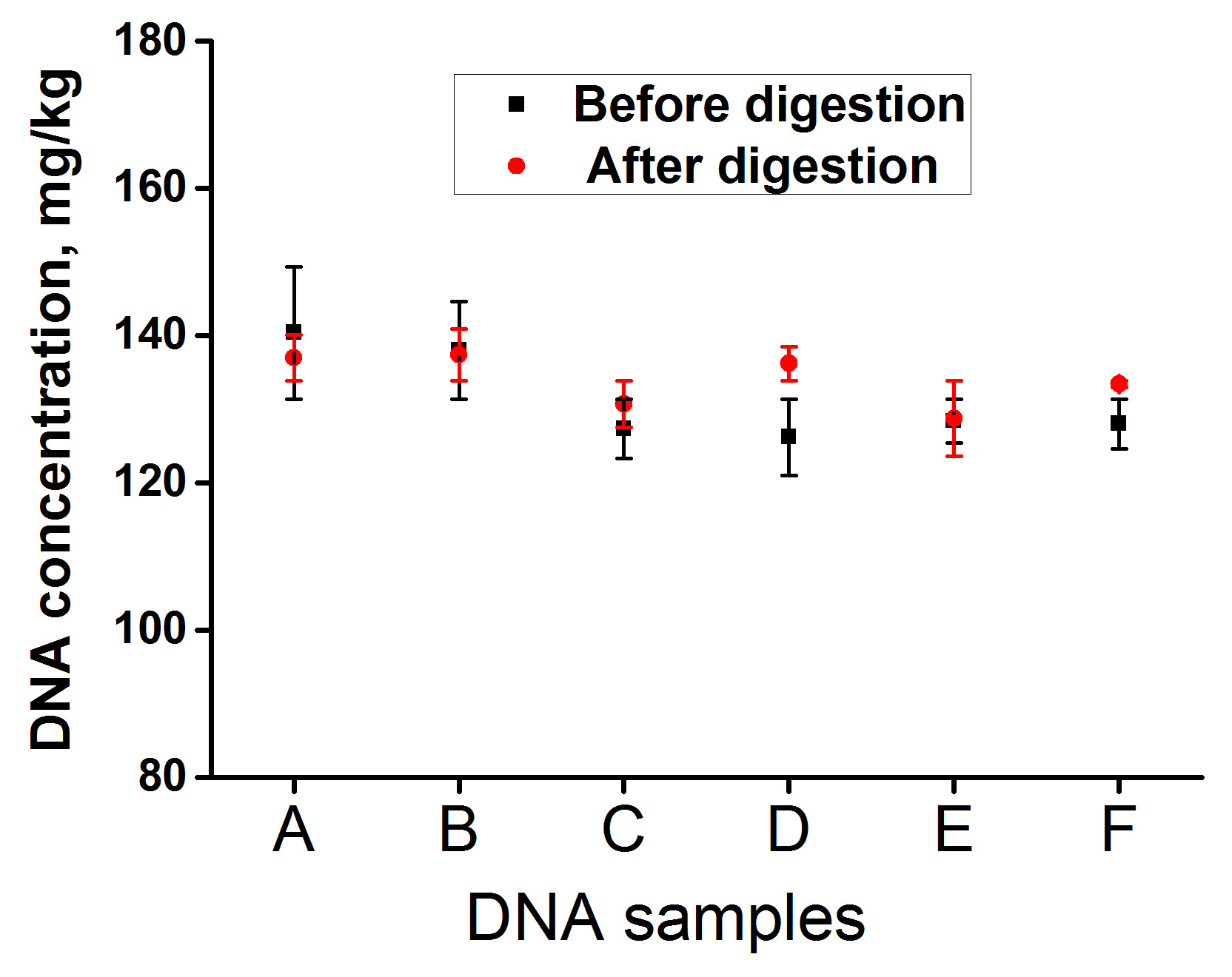


**Fig. S4** A DNA certified reference material (GBW(E)100274, certified concentration 137±11 mg/kg) was separated into 6 samples (A, B, C, D, E, F) and analyzed by HR-ICP-MS, both before digestion and after digestion. The average results were 131±6 before digestion and 130±1 after digestion, and the difference them was insignificant due to t-test.

**Fig. S5** As a positive control, we present here the quantification results of a certified reference material (IRMM-449) with a certified concentration of 13±7 ng/μL. For all the 3 methods in our work, good consistence was achieved and all the standard deviations were below the uncertainty of IRMM-449.

**Table S1** Homogeneity analysis result of the plasmids by UV. According to ANOVA*, F_cal_* is the *F* test value, *F_0.05_*(*m,n*) is the critical values at 95% confidence, where *m* is the sample number and *n* is the degree of freedom of the analysis data. *MS_between_* is the mean square of the analysis data of between-bottle analysis, and *MS_within_* is the mean square of the analysis data of within-bottle analysis. Good homogeneity is demonstrated when *F_cal_<F_0.05_*(*m,n*). ***u_bb,rel_*** is the relative uncertainty of inhomogeneity

| **Sample pFliC**  **(*m*=15)** | **Concentration of subsamples (*n*) (ng/μL)** | | |
| --- | --- | --- | --- |
|  | **1** | **2** | **3** |
| 1 | 71.1 | 72.6 | 73.4 |
| 2  3 | 71.4  73.3 | 71.3  71.9 | 71.7  72 |
| 4 | 71.1 | 72.8 | 70.1 |
| 5 | 71.1 | 71.7 | 72.2 |
| 6 | 72.3 | 71 | 74.5 |
| 7 | 71.5 | 72.1 | 72.9 |
| 8 | 71.5 | 72.9 | 71.4 |
| 9 | 72.3 | 70.7 | 73.1 |
| 10 | 72.9 | 70.7 | 70.6 |
| 11 | 72.2 | 72.4 | 72.5 |
| 12 | 71 | 71.4 | 72.4 |
| 13 | 71.9 | 71.1 | 72.4 |
| 14 | 72.2 | 72.3 | 72.4 |
| 15 | 72.2 | 71.3 | 71.2 |
| *MS _between_* | 7.30 | | |
| *MS _within_* | 25.96 | | |
| F*_cal_* | 0.603 | | |
| *F*_0.05_(14, 30) | 2.04 | | |
| ***u_bb,rel_*** | **0.%** | | |
| **Sample pStx1**  **(*m*=15)** | **Concentration of subsamples (*n*) (ng/μL)** | | |
|  | **1** | **2** | **3** |
| 1 | 59.8 | 60.4 | 62 |
| 2 | 59.7 | 60.1 | 60.3 |
| 3 | 60.1 | 61.6 | 61.9 |
| 4 | 60.4 | 60.5 | 60.6 |
| 5 | 60.1 | 60.2 | 60.7 |
| 6 | 62.0 | 59.7 | 60.5 |
| 7 | 60.9 | 60.5 | 60.9 |
| 8 | 59.4 | 59.0 | 62.0 |
| 9 | 60.8 | 62 | 60.6 |
| 10 | 59.3 | 59.1 | 59.6 |
| 11 | 60.3 | 61.8 | 59.7 |
| 12 | 60.5 | 60.7 | 60.6 |
| 13 | 59.5 | 59.7 | 61.3 |
| 14 | 60.9 | 59.9 | 61.4 |
| 15 | 58.7 | 60.9 | 59.2 |
| *MS _between_* | **11.26** | | |
| *MS _within_* | **22.41** | | |
| F*_cal_* | **1.077** | | |
| *F*_0.05_(14, 30) | **2.04** | | |
| ***u_bb,rel_*** | **0.4%** | | |
| **Sample pStx2**  **(*m*=15)** | **Concentration of subsamples (*n*) (ng/μL)** | | |
|  | **1** | **2** | **3** |
| 1 | 68.9 | 70.7 | 69.1 |
| 2 | 68.8 | 65.8 | 69 |
| 3 | 67.7 | 68.2 | 69.3 |
| 4 | 67.2 | 67.2 | 69.7 |
| 5 | 66.8 | 69.7 | 69.8 |
| 6 | 69.4 | 67.8 | 70.4 |
| 7 | 66.7 | 68.5 | 68.4 |
| 8 | 68 | 66.1 | 68.3 |
| 9 | 66.8 | 67.6 | 68.8 |
| 10 | 67.8 | 67.8 | 68.7 |
| 11 | 65.7 | 66.7 | 67.8 |
| 12 | 67.8 | 68.5 | 68.7 |
| 13 | 68.9 | 68 | 70.8 |
| 14 | 68.8 | 68 | 66.1 |
| 15 | 68.6 | 69.4 | 65.6 |
| *MS_between_* | **23.7** | | |
| *MS_within_* | **49.2** | | |
| F*_cal_* | **1.03** | | |
| *F*_0.05_(14, 30) | **2.04** | | |
| ***u_bb,rel_*** | **0.6%** | | |

**Table S2,** Short-term stability analysis of plasmid DNA RMs by UV (storage conditions: 4 °C). *β_1_* is the slope of stability curve**,** *s*(*β_1_*) is the standard deviation of the slope

| **Storage time (days)** | **pFliC (ng/μL)** | **pStx1 (ng/μL)** | **pStx2 (ng/μL)** |
| --- | --- | --- | --- |
| 0 | 72.80 | 62.08 | 70.22 |
| 1 | 73.27 | 60.97 | 69.25 |
| 3 | 72.73 | 60.57 | 69.17 |
| 7 | 72.27 | 61.87 | 69.17 |
| 15 | 72.17 | 62.20 | 70.30 |
| *β_1_* | 0.084 | 0.059 | 0.035 |
| *S(β_1_)* | 0.051 | 0.060 | 0.051 |
| ***t_0.95,n-2_*** | **12.7** | **12.7** | **12.7** |
| **Conclusion** | **Stable** | **Stable** | **Stable** |

**Table S3** Long-term stability analysis of the plasmid DNA RMs by UV. (storage conditions: -20 °C). *β_1_* is the slope of stability curve**,** *s(β_1_)* is the standard deviation of the slope**,** *n* is the number of analysis replicates, ***u_S,rel_*** is the relative uncertainty from instability

| **Storage time (months)** | **pFliC (ng/μL)** | **pStx1 (ng/μL)** | **pStx2 (ng/μL)** |
| --- | --- | --- | --- |
| 0 | 71.90 | 62.08 | 68.61 |
| 0.5 | 71.37 | 62.43 | 69.67 |
| 1 | 68.67 | 59.37 | 67.67 |
| 2 | 71.17 | 60.73 | 69.30 |
| 4 | 68.07 | 57.87 | 67.33 |
| 6 | 69.00 | 58.33 | 67.23 |
| 9 | 69.03 | 59.98 | 69.27 |
| 12 | 68.38 | 58.48 | 69.82 |
| *β_1_* | 0.223 | 0.116 | 0.060 |
| *S(β_1_)* | 0.109 | 0.088 | 0.096 |
| ***t_0.95,n-2_*** | **12.7** | **12.7** | **12.7** |
| **Conclusion** | **Stable** | **Stable** | **Stable** |
| ***u_S,rel_*** | **1.9%** | **1.8%** | **1.8%** |

**Table S5** the quantification results and uncertainty evaluation of three different methods.

| pFliC (10^10^copies/μL) | | | | |
| --- | --- | --- | --- | --- |
| *n* | | UV | HR-ICP-MS | dPCR |
| 1 | 1.59 | | 1.58 | 1.70 |
| 2 | 1.59 | | 1.64 | 1.65 |
| 3 | 1.63 | | 1.65 | 1.56 |
| 4 | 1.59 | | 1.59 | 1.55 |
| 5 | 1.59 | | 1.63 | 1.56 |
| 6 | 1.61 | | 1.54 | 1.68 |
| 7 | 1.59 | | 1.58 | 1.64 |
| 8 | 1.59 | | 1.50 | 1.59 |
| Average | 1.60 | | 1.59 | 1.62 |
| Relative uncertainty (%) | 0.4 | | 1.4 | 2.1 |

| pStx1 (10^10^copies/μL) | | | | |
| --- | --- | --- | --- | --- |
| n | | UV | HR-ICP-MS | dPCR |
| 1 | 1.51 | | 1.79 | 1.45 |
| 2 | 1.51 | | 1.86 | 1.46 |
| 3 | 1.52 | | 1.87 | 1.55 |
| 4 | 1.53 | | 1.81 | 1.57 |
| 5 | 1.52 | | 1.85 | 1.57 |
| 6 | 1.57 | | 1.75 | 1.43 |
| 7 | 1.54 | | 1.79 | 1.45 |
| 8 | 1.50 | | 1.70 | 1.47 |
| Average | 1.53 | | 1.80 | 1.50 |
| Relative uncertainty (%) | 0.5 | | 1.7 | 1.9 |

| pStx2(10^10^copies/μL) | | | | |
| --- | --- | --- | --- | --- |
| *n* | | UV | HR-ICP-MS | dPCR |
| 1 | 1.74 | | 1.66 | 1.69 |
| 2 | 1.74 | | 1.75 | 1.81 |
| 3 | 1.71 | | 1.65 | 1.72 |
| 4 | 1.70 | | 1.69 | 1.67 |
| 5 | 1.69 | | 1.74 | 1.74 |
| 6 | 1.75 | | 1.64 | 1.69 |
| 7 | 1.69 | | 1.62 | 1.75 |
| 8 | 1.72 | | 1.67 | 1.69 |
| Average | 1.72 | | 1.68 | 1.72 |
| Relative uncertainty (%) | 0.5 | | 1.5 | 1.9 |

**Table S6** The uncertainty sources of the concentration of three RMs (10^10^copies/μL)

| Uncertainty sources | | pFliC | pStx1 | pStx2 |
| --- | --- | --- | --- | --- |
| $\overline{X}$ | 1.60 | | 1.53 | 1.70 |
| *u_c_* | 0.041 | | 0.039 | 0.042 |
| $u_{bb}$ | 0.006 | | 0.006 | 0.010 |
| $u_{s}$ | 0.030 | | 0.028 | 0.031 |
| *u* | 0.052 | | 0.048 | 0.053 |
| *U* | 0.10 | | 0.10 | 0.11 |

(*u_c_* is the uncertainty from quantification, including UV, HR-ICP-MS and dPCR, $u_{bb}$ is the uncertainty from inhomogeneity, $u_{s}$ is the uncertainty from instability. *u* is the combined uncertainty, and *U* is  the expanded uncertainty (*k*=2).)
